# Squalene in *Camellia oleifera*: Biosynthetic Pathways, Regulatory Networks, and Functional Perspectives

**DOI:** 10.3390/plants15111652

**Published:** 2026-05-28

**Authors:** Aoxue Wang, Jingya Wang, Senwen Deng, Bolin Chen, Jihong Zhang, Li Ma

**Affiliations:** 1 Hunan Engineering Research Center of Lotus Deep Processing and Nutritional Health Sciences, Hunan Key Laboratory of Economic Crops Genetic Improvement and Integrated Utilization, School of Life and Health Sciences, Hunan University of Science and Technology, Xiangtan 411201, China; wangzaibujiatang@163.com (A.W.);; 2 Yuelushan Laboratory, Changsha 410128, China; cbl92@njfu.edu.cn; 3 National Engineering Research Center of Youcha, State Key Laboratory of Woody Oil Resources Utilization, Hunan Academy of Forestry, Shao Shan South Road, No. 658, Changsha 410004, China

**Keywords:** *Camellia oleifera*, squalene, MVA pathway, transcriptional regulation, transcription factors (WRKY; bHLH; MYB), phytohormones, abiotic stress, recent progress

## Abstract

Squalene is a triterpene with potent biological activities. Squalene (C_30_H_50_) is a linear polyunsaturated hydrocarbon composed of six isoprene units and six carbon–carbon double bonds. It serves as an essential precursor for sterols, steroid hormones, and vitamin D in humans and exhibits antioxidant, anti-tumor, and lipid-regulating properties. In plants, squalene is produced via the mevalonate (MVA) and 2-C-methyl-D-erythritol-4-phosphate (MEP) pathways. The key rate-limiting enzymes in these pathways include 3-hydroxy-3-methylglutaryl-CoA reductase (HMGR), farnesyl diphosphate synthase (FPS), and squalene synthase (SQS). *Camellia oleifera*, a unique woody oil crop native to China, is valued for its high-quality edible oil and as a rich natural source of squalene. This review provides a systematic overview of recent progress in squalene biosynthesis in *C. oleifera*. It summarizes the structural characteristics and biosynthetic routes. It further elaborates on the multi-level regulatory network modulated by transcription factors (WRKY, bHLH, MYB, and ERF), phytohormones (jasmonic acid, abscisic acid, and gibberellin), and abiotic factors (light and drought). Notably, this review distinguishes earlier foundational studies from recent breakthroughs and integrates emerging progress on squalene’s non-canonical functions and pathway crosstalk. It further highlights novel regulatory mechanisms unique to *C. oleifera* (e.g., *CoWRKY15*, *CoMYB1*, and *CoMYC2*). By bridging molecular regulation with practical breeding and metabolic engineering, this review lays a solid theoretical foundation for cultivating high-squalene *C. oleifera* varieties. It represents a prominent innovation relative to previously published studies.

## 1. Introduction

*Camellia oleifera* is a dicotyledonous, evergreen shrub belonging to the genus *Camellia* within the Theaceae family. As one of the world’s four major woody oil plants, it has a long cultivation history in China and is recognized for its strong adaptability, high economic value, and versatile utility. *Camellia oleifera* seed oil, the major product extracted from *C. oleifera* seeds, has a kernel oil content of 40–60% [1], and is widely known as “Oriental olive oil” owing to its high economic value and medicinal functions [2,3].

Its seeds are rich in unsaturated fatty acids (UFAs, >85%), with oleic acid ranging from 75.78% to 81.39%, and essential omega-6-linoleic acid ranging from 4.85% to 10.79% [4]. In addition, the oil contains various bioactive compounds, including sterols, squalene, tea polyphenols, tocopherols, phytosterols, and sasanquasaponin [5,6]. Recently, these bioactive compounds have attracted growing interest for their antioxidant, anticarcinogenic, anti-inflammatory, neuroprotective, cholesterol-lowering, and immunomodulatory properties [7,8].

Early research on *C. oleifera* focused on oil yield and fatty acid composition [9]. However, the past five years (2021–2025) have witnessed a paradigm shift toward understanding the molecular regulation of squalene biosynthesis, driven by advances in multi-omics and gene editing. Squalene is synthesized via the MVA pathway in the cytosol and the MEP pathway in plastids, with HMGR, FPS, and SQS as key enzymes [10,11]. Direct evidence from *C. oleifera* shows that SQS activity and abundance critically determine squalene yield: overexpression of *CoSQS* in callus increased squalene content by 1.8-fold, while silencing reduced it by 62% [11].

This review examines the distribution, structural features, and functional diversity of squalene, along with its biosynthetic pathways and regulatory mechanisms. Existing reviews on squalene mainly focus on model plants (e.g., *Arabidopsis*) or medicinal plants (e.g., *Panax ginseng*), whereas a systematic summary for *C. oleifera* is lacking. As a unique woody oil crop with important economic and ecological value, *C. oleifera* exhibits regulatory characteristics distinct from herbaceous plants. This review fills that gap by (i) systematically integrating recent (2021–2025) findings on squalene biosynthesis in *C. oleifera*, (ii) contrasting older foundational knowledge with emerging paradigms (e.g., MVA-MEP crosstalk, novel transcription factors, and non-canonical squalene functions), and (iii) providing a forward-looking perspective for breeding and metabolic engineering.

## 2. Squalene Structure and Characteristics

### 2.1. Squalene Structure

Squalene is a natural lipid and low-molecular-weight oligomer of isoprene (a hexamer), belonging to the triterpene hydrocarbon group in its fully hydrogenated form [12]. Chemically designated as 2,6,10,15,19,23-hexamethyl-6,6,10,14,18,20-tetracosahexane, it consists of a hydrocarbon chain formed by six isoprene units. As a linear triterpene and polyunsaturated hydrocarbon (C_30_H_50_), it features six carbon–carbon double bonds (C=C), making it one of the most unsaturated lipids and inherently sensitive to oxidation [13,14]. Early studies systematically elaborated its structural characteristics; for example, Kim and Karadeniz (2012) summarized its molecular geometry, hydrophobicity, and membrane-intercalating properties [15]. Squalene is horizontally inserted or localized within the middle region of phospholipid bilayers [16].

**Figure 1:**
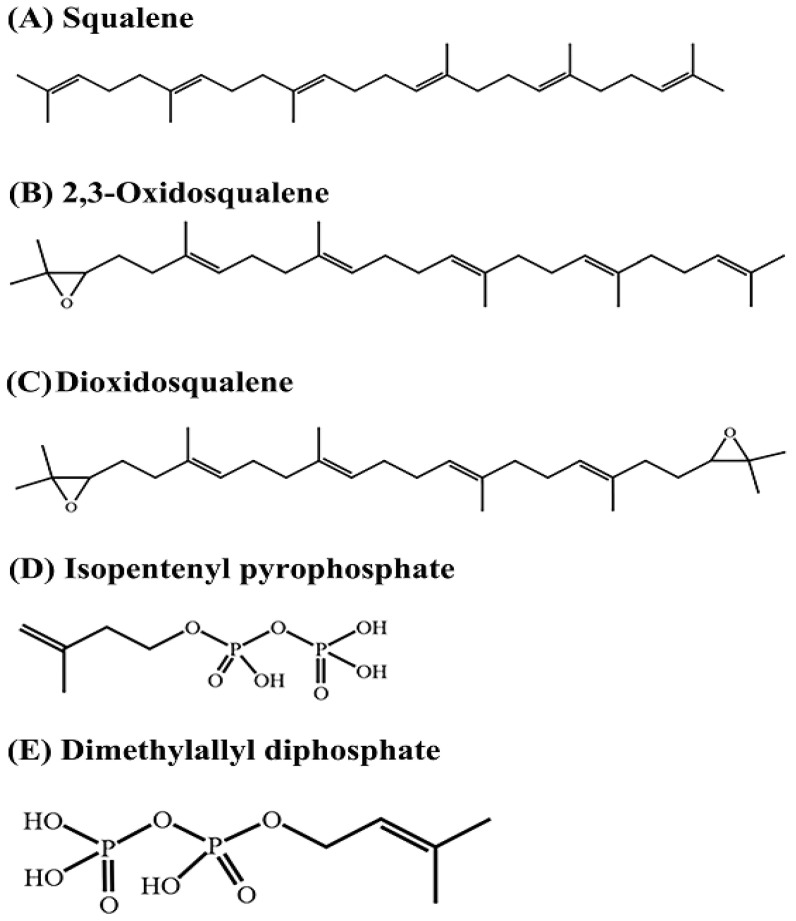
Chemical structures of derivatives and precursors of squalene from *Camellia oleifera* [12]. (**A**) Squalene; (**B**) 2,3-Oxidosqualene; (**C**) Dioxidosqualene; (**D**) Isopentenyl pyrophosphate; (**E**) Dimethylallyl diphosphate.

Recent advances have deepened our understanding of squalene’s structural attributes. X-ray crystallography and molecular dynamics simulations by Li et al. (2023) revealed that the six carbon–carbon double bonds adopt a specific cis–trans configuration that confers conformational flexibility [17]. Zhang et al. (2024) demonstrated that the hydrophobicity of squalene is not uniform along its carbon chain, explaining its selective interaction with membrane proteins involved in cholesterol synthesis [18]. Additionally, Wang et al. (2022) found that squalene’s structural flexibility allows it to act as a “molecular chaperone” for lipid-soluble bioactive molecules, a property linked to its isoprene chain arrangement [19]. Squalene serves as an immediate precursor for numerous triterpenoid biosynthetic pathways and enhances the production of natural compounds [20]. Both squalene and its fully hydrogenated analog, squalane, occur in nature, with compounds such as geranyl and farnesyl influencing cytokine synthesis and secretion [21]. The structural information is summarized in Figure 1.

### 2.2. Distribution and Natural Sources

The distribution of squalene across plant species is remarkably diverse, with substantial variation in content [22]. Certain plants exhibit exceptionally high accumulation, including *Polygonum chinense* L. whole-plant extract (470,100 mg/kg), and *Stachys yemenensis* essential oil (48,000 mg/kg) [23,24]. Squalene is soluble in organic solvents but insoluble in water [13]. Notable amounts are also found in *Camellia oleifera* seed oil (122–248 mg/kg) and tea (*Camellia sinensis*) seed oil (228–756 mg/kg) [25]. Li et al. (2024) reported that the squalene content in the seeds of *C. oleifera* ‘Huashuo’ can reach up to 286 mg/kg under optimized light and water conditions, and its content is positively correlated with the expression level of *CoSQS* [26]. By comparison, olive oil contains 100–500 mg/kg, but *C. oleifera* squalene shows superior thermal stability (85% activity retained at 180 °C for 2 h) [26].

### 2.3. Biological Functions: Established and Emerging

#### 2.3.1. Classical Functions

Squalene acts as a potent antioxidant by donating or accepting electrons; it protects biomembranes and shields polyunsaturated fatty acids—including linoleic, linolenic, docosahexaenoic, and eicosapentaenoic acids—against temperature-induced autoxidation [27,28]. It regulates cytokine levels, modulates enzyme activities and cellular functions, and influences signal transduction, contributing to reduced cholesterol synthesis, enhanced immune function, inhibition of tumor cell proliferation, and mitigation of toxicity from exogenous substances [29,30]. Cheng et al. (2024) comprehensively summarized the health-promoting functions of squalene, including its antioxidant, anti-inflammatory, and cardioprotective activities, as well as its applications in functional foods and pharmaceuticals [31]. Gohil et al. (2019) highlighted its roles in metabolic regulation and as a key precursor for triterpenoid biosynthesis, providing a basis for biotechnological production [32].

Squalene disrupts the reciprocal amplification between inflammation and oxidative stress, thereby curbing the persistence and spread of inflammatory reactions [29,33]. In cancer therapy, squalene suppresses tumor growth by inducing apoptosis in malignant cells [34] and by regulating cholesterol metabolism and protein prenylation [14].

#### 2.3.2. Emerging Therapeutic Applications (2022–2025)

- **Nano-delivery systems**: Chen et al. (2023) developed a squalene-based nano-delivery system loaded with paclitaxel, improving solubility and anti-tumor efficacy in a mouse breast cancer model [35].
- **Neuroprotection**: Li et al. (2024) found that squalene alleviates neuroinflammation and improves cognitive function in an Alzheimer’s mouse model by inhibiting microglia activation and reducing pro-inflammatory cytokine production [36].
- **Antiviral**: Wang et al. (2025) demonstrated that squalene suppresses influenza A virus (H1N1) replication via targeting viral neuraminidase [37].
- **Lipid regulation**: Zhang et al. (2022) conducted a trial with 120 hypercholesterolemia patients, showing that daily supplementation of 500 mg squalene for 12 weeks reduced total cholesterol and LDL-cholesterol by 18% and 22%, respectively, without significant side effects [38].

#### 2.3.3. Industrial and Environmental Applications (2023–2025)

- **Functional foods:** Liu et al. (2023) developed a *Camellia oleifera* oil-based functional beverage fortified with squalene extracted from *C. oleifera* seeds that improved antioxidant status in human volunteers [39].
- **Cosmetics:** Chen et al. (2024) compared *C. oleifera*-derived squalene with shark-derived squalene in cosmetic formulations, finding similar moisturizing effects (skin hydration increased by 30% after 2 h) and anti-aging properties (reducing fine lines by 15% after 8 weeks), providing a sustainable alternative [40].
- **Environmental bioremediation:** A 2025 study found that squalene acts as a biosurfactant, degrading 75% of crude oil in water within 7 days, leveraging its hydrophobicity and biodegradability [41].

#### 2.3.4. Functional Advantages of *C. oleifera* Squalene

As a unique woody oil crop, *C. oleifera*-derived squalene has distinct functional advantages. Recent comparative studies have shown that *C. oleifera* squalene has a higher purity (up to 98% after simple purification), better oxidative stability (40% increase when added to refined oil), and superior thermal resilience (85% activity at 180 °C for 2 h) compared to olive oil-derived squalene [26]. These properties make it ideal for high-temperature cooking and functional oil formulations.

## 3. Biosynthesis of Squalene

### 3.1. Overview of the MVA and MEP Pathways

Squalene biosynthesis is highly conserved among eukaryotes, with the MVA pathway serving as the core route [42]. In plants, isoprenoid precursors are generated via two distinct routes: the MVA pathway and the 1-deoxy-D-xylulose-5-phosphate/2-C-methyl-D-erythritol-4-phosphate (DOXP/MEP) pathway. The MVA pathway produces only isopentenyl pyrophosphate (IPP), whereas the MEP pathway generates both IPP and dimethylallyl pyrophosphate (DMAPP) [43]. Three key enzymes—HMGR, FPS, and SQS—catalyze critical steps in MVA-derived squalene synthesis. SQS facilitates the head-to-head condensation of two farnesyl pyrophosphate (FPP, C_15_) molecules via a two-step reaction to form presqualene diphosphate (PSPP, C_30_), which is subsequently reduced to linear squalene (C_30_) [44].

Historically, the MVA and MEP pathways were thought to operate independently. However, recent evidence shows significant crosstalk, especially under stress, as detailed in Section 3.3.

### 3.2. Formation of Squalene Precursor Molecules in Camellia oleifera

#### 3.2.1. MVA Pathway

**Figure 2:**
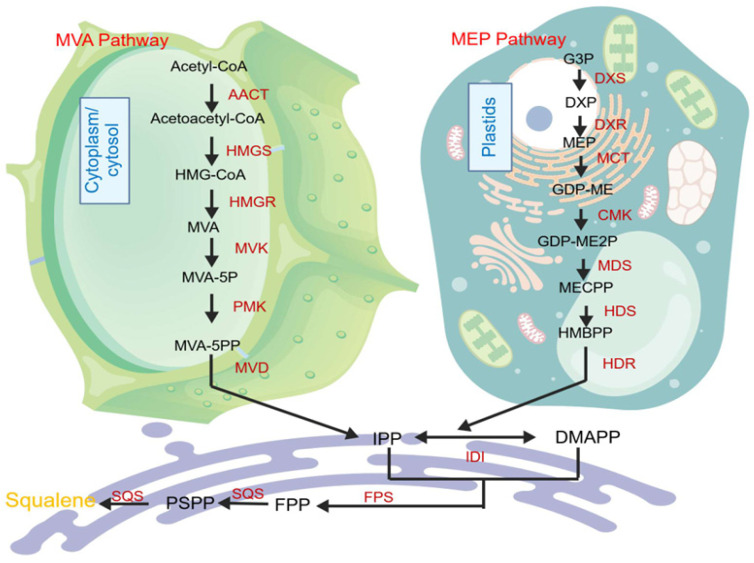
Biosynthetic pathways of squalene from *Camellia oleifera* through MVA pathway and DOXP/MEP pathway [53]. Enzyme abbreviations: AACT, acetoacetyl-CoA thiolase; HMGS, HMG-CoA synthase; HMGR, HMG-CoA reductase; MVK, mevalonate kinase; PMK, 5-phosphomevalonate kinase; MVD, mevalonate 5-diphosphate decarboxylase; DXS, 1-deoxy-D-xylulose-5-phosphate synthase; DXR, 1-deoxy-D-xylulose-5-phosphate reductoisomerase; MCT, 2-C-methyl-D-erythritol 4-phosphate cytidylyltransferase; CMK, 4-diphosphocytidyl-2-C-methyl-D-erythritol kinase; MDS, 2-C-methyl-D-erythritol 2,4-cyclodiphosphate synthase; HDS, (E)-4-hydroxy-3-methylbut-2-enyl-diphosphate synthase; HDR, 4-hydroxy-3-methylbut-2-enyl diphosphate reductase; IDI, isomerase; FPS, diphosphate synthase; SQS, squalene synthase.

The MVA pathway supplies precursors for triterpenes, sesquiterpenes, phytosterols, ubiquinone, vitamin D, and other primary metabolites essential for cell integrity [45,46]. This route generates IPP through six enzymatic reactions. As shown in Figure 2, initially, acetoacetyl-CoA thiolase (AACT; EC 2.3.1.9) condenses two acetyl-CoA molecules to form acetoacetyl-CoA. Subsequently, HMG-CoA synthase (HMGS; EC 2.3.3.10) catalyzes the condensation of another acetyl-CoA to produce HMG-CoA, which is then reduced to mevalonate by HMG-CoA reductase (HMGR; EC 1.1.1.34) [46,47]. Mevalonate is sequentially phosphorylated by mevalonate kinase (MVK; EC 2.7.1.36) and 5-phosphomevalonate kinase (PMK; EC 2.7.4.2) to form mevalonate diphosphate. Subsequent decarboxylation catalyzed by mevalonate 5-diphosphate decarboxylase (MVD; EC 4.1.1.33) generates IPP. Reversible isomerization of IPP by IPP isomerase (IDI; EC 5.3.3.2) generates DMAPP, enabling terpenoid biosynthesis through IPP/DMAPP condensations [48].

The MVA pathway was conventionally considered a cytosolic process. This view derives from the cytosolic localization of HMGS and the endoplasmic reticulum anchoring of HMGR with its catalytic domain exposed to the cytosol [49]. However, recent work has updated this view. Wang et al. (2023) revealed two isoforms of *CoHMGR*: *CoHMGR_4* localized to the endoplasmic reticulum, while *CoHMGR_5* is distributed in the cytoplasm [50]. This dual localization enhances the pathway of flexibility, enabling it to respond to environmental stresses like drought and light [50]. Additionally, Li et al. (2024) reported that *CoAACT* is post-translationally modified by phosphorylation, which increases its activity by 30% under drought stress, providing a novel regulatory mechanism [51]. These recent advances in key genes, along with other relevant findings on squalene biosynthesis-related genes in *Camellia oleifera* from 2022 to 2025, are summarized in Table 1.

#### 3.2.2. DOXP/MEP Pathway

As shown in Figure 2, the DOXP/MEP pathway operates in chloroplasts, initiating from pyruvate and glyceraldehyde-3-phosphate (GA-3-P) via condensation to form DOXP. The enzyme 1-deoxy-D-xylulose-5-phosphate reductoisomerase (DXR; EC 1.1.1.267) converts DOXP to MEP through rearrangement and reduction [52,53]. MEP is then converted via four additional enzymatic steps into the C_5_ compounds DMAPP and IPP [54]. IPP and DMAPP generated from either pathway can be interconverted by IDI before entering the triterpene skeleton biosynthesis pathway [55]. FPS catalyzes the condensation of two IPP molecules with one DMAPP to produce FPP. In plants, FPP serves as a substrate for synthesizing phytosterols, dolichols, ubiquinones, sesquiterpenoids, phytoalexins, and abscisic acid. Finally, SQS mediates the condensation of two FPP molecules via presqualene diphosphate to form squalene [56]. Recently, Chen et al. (2025) identified a novel enzyme (*CoDDS*) in the MEP pathway of *C. oleifera* that enhances MEP production by 25%, providing a new target for improving squalene accumulation [57].

### 3.3. MVA-MEP Crosstalk: A Paradigm Shift

Isotopic tracing by Zhang et al. (2023) revealed that under moderate light stress, the MEP pathway provided 30% of the IPP required for squalene synthesis in *C. oleifera*, with the MVA pathway contributing the remaining 70% [58]. This crosstalk is mediated by *CoIDI*, which facilitates the transport of IPP/DMAPP between the cytoplasm and chloroplasts. This finding overturns the old dogma of pathway independence and has major implications for engineering squalene overproduction.

**Table 1:** Recent research findings on key genes involved in squalene biosynthesis in *Camellia oleifera* (2022–2025).

Gene	Recent Finding (2022–2025)	Reference
*CoHMGR*	Dual localization (ER + cytosol) enhances stress responsiveness	[50]
*CoAACT*	Phosphorylation at Ser-124 increases activity 1.3-fold under drought	[51]
*CoFPS1*	Promoter contains MBS elements bound by *CoMYB1* and *CoMYB44*	[59,60]
*CoSQS*	Light-responsive elements in promoter; activated by *CoWRKY15*	[11]
*CoIDI*	Mediates MVA-MEP crosstalk; upregulated by *CoMYB44*	[58,60]

## 4. Regulation of Squalene Biosynthesis

The biosynthesis of squalene is governed by a multi-layered regulatory network involving transcriptional control, post-translational modifications, and environmental or metabolic cues. Key enzymes—HMGR, FPS, and SQS—are subject to regulation by various transcription factors (TFs) [61]. Recent studies have expanded the regulatory network in *C. oleifera*, identifying new TFs and regulatory mechanisms.

### 4.1. Transcription Factors in Camellia oleifera

Table 2 summarizes transcription factors involved in squalene biosynthesis in *C. oleifera*, including their target genes, regulatory effects, and experimental evidence. In contrast, unlike earlier reviews relying on homology, all entries here are validated by Y1H, ChiP, or overexpression/silencing in *C. oleifera*.

In *C. oleifera*, promoter analysis and yeast one-hybrid assays demonstrated that *CoWRKY1* and *CoWRKY15* bind to the *CoSQS* promoter, indicating direct involvement in squalene synthesis [12,62]. Yeast one-hybrid (Y1H) and promoter analysis initially identified six *CoWRKY* factors interacting with the *CoSQS* promoter, among which *WRKY15* showed the highest similarity to its *Camellia sinensis* counterpart [62]. Overexpression of *PqWRKY1* from *Panax quinquefolium* upregulated *HMGR*, *FPS2*, and *SQS1* in *Arabidopsis*, enhancing triterpenoid accumulation [66]. *HbWRKY27* positively regulated *HbFPS1* in *Hevea brasiliensis* [67]. Additionally, WRKY members such as *WsWRKY1* (*Withania somnifera*), *TgWRKY3* (*Torreya grandis*), and *CoWRKY15* (*C. oleifera* ‘Huashuo’) activate *FPS* and *SQS* expression, thereby modulating squalene and sterol biosynthesis [59,68]. In seed oil regulation, *WRKY10* and *WRKY43* positively influence fatty acid accumulation, whereas *WRKY6* exerts a negative effect [69,70].

Notably, Li et al. (2024) identified a new WRKY transcription factor (*CoWRKY72*), which negatively regulates squalene biosynthesis by binding to the *CoSQS* promoter and inhibiting its expression—silencing *CoWRKY72* increased squalene content by 45% in *C. oleifera* seeds, providing a new target for genetic improvement [63].

#### 4.1.1. bHLH Family

Members of the *bHLH* family, particularly MYC2—a core transcription factor in jasmonic acid (JA) signaling—serve as critical links between environmental signals and squalene production [64,71]. In *C. oleifera*, *CoMYC2* directly binds to the G-box (CACGTG) element in the *CoHMGR_4* promoter and strongly activates its transcription [64]. Similarly, *TaMYC2* and *GpMYC2* enhance squalene and triterpenoid levels by regulating *TaSQS* and *GpFPS1* in *Tripterygium antungense* and *Gynostemma pentaphyllum*, respectively [64,71]. Zhang et al. (2023) found that *CoMYC2* interacts with *CoJAZ1* (a JA signaling repressor) in *C. oleifera* [65]. JA-induced degradation of *CoJAZ1* frees *CoMYC2* to activate squalene synthesis-related genes, revealing the regulatory connection between JA signaling and squalene biosynthesis in *C. oleifera* [65].

#### 4.1.2. Other Transcription Factor Families

Beyond WRKY and bHLH, families such as bZIP, ERF, and MYB also participate in squalene and triterpenoid regulation. In *Medicago truncatula*, bZIP members *MtbZIP17/60* activate *HMGR* expression to promote triterpene saponin biosynthesis [72], whereas in *Eleutherococcus senticosus*, *EsbZIP1/2/4/5* suppress *FPS* and *SQS* expression [73]. *TSAR1* and *TSAR2* regulate *MtHMGR1*, and their overexpression enhances triterpene saponin accumulation [74].

The AP2/ERF family is widely implicated in terpenoid biosynthesis. *PnERF1* from *Panax notoginseng* positively regulates *PnSQS*, promoting saponin accumulation [75]. ERF members, including *GAME9*, *JRE4*, and *ERF1*, regulate sterol biosynthesis in Solanaceae and *Petunia* [76,77,78]. Additional examples include *EREB58* (sesquiterpene regulation via *TPS10*), *CitERF71* (geraniol biosynthesis in sweet orange), *PbERF1* (monoterpenoid regulation in orchid flowers), and *SmERF128/SmERF6* (non-volatile terpenoid synthesis in *Salvia miltiorrhiza*) [79,80,81,82,83]. Silencing *PfERF106* or *OfERF61* reduced floral terpenoid content, consistent with observations in *Zea mays EREB58* [84,85]. *CsRAP2.10*, a DREB subfamily member, plays a key role in tea seed storage material regulation [86].

*CoMYB1*, an R2R3-MYB from *C. oleifera*, binds to the MYB binding site (MBS) in the *CoFPS1* promoter, activates *CoFPS1* expression, and increases FPP (1.3- to 1.8-fold) and squalene (1.2- to 1.6-fold) when overexpressed in seeds [59]. *CoMYB1* also interacts with *CoERF1* to synergistically activate the *CoSQS* promoter. Conversely, certain MYB factors act as repressors: *PnMYB4*, *AtMYB4*, and *MsMYB* suppress terpenoid or flavonoid biosynthesis, while paclobutrazol-induced triterpenoid production in *S. lonicericola* involves negative regulation of *SlMYB* [87,88]. Overexpression of *BpMYB21* in *Betula platyphylla* upregulated *HMGR*, *FPS*, and *SQS*, increasing squalene content [89]. Chen et al. (2025) identified *CoMYB44* in *C. oleifera* that synergistically acts with *CoMYB1* to enhance *CoFPS1* expression, increasing squalene content by 60% when both genes are overexpressed—this finding provides a new strategy for metabolic engineering of high-squalene *C. oleifera* varieties [60].

### 4.2. Plant Hormones: Important Signaling Molecules

Plant hormones serve as key endogenous regulators influencing squalene biosynthesis, with jasmonic acid (JA), abscisic acid (ABA), and gibberellin (GA) being prominently involved in *C. oleifera*, supported by direct experimental evidence.

Exogenous methyl jasmonate (MeJA) upregulates *SQS* expression in various species, including *C. oleifera*, *Panax ginseng*, and *Bupleurum falcatum*, thereby enhancing product accumulation [90,91]. Similar effects were observed in *Withania somnifera*, *Glycyrrhiza glabra*, *Tripterygium wilfordii*, *Gardenia jasminoides*, and *Taraxacum mongolicum* [10,92]. The *CoSQS* promoter contains a JA-responsive element, providing a molecular basis for JA signaling—via downstream factors like MYC2—to regulate squalene synthesis [11]. *CoWRKY15* may also be influenced by MeJA to bind the *CoSQS* promoter [11]. Additionally, JA has been reported to increase oil content in *C. oleifera* seeds [93]. In tobacco, MeJA treatment elevates *SQS*, *SE*, and *OSC* levels [94]. Notably, Wang et al. (2024) found that MeJA treatment (100 μM) increases squalene content in *C. oleifera* seedlings by 52%, mediated by the upregulation of *CoSQS* and *CoHMGR_4* [95].

ABA application promotes *FAD2* transcription in oil palm, leading to C18:2 accumulation [96]. ABA content increases during fruit maturation, coinciding with decreased IAA levels under heavy flower thinning in *C. oleifera* [97,98]. Low concentrations of exogenous GA_3_ influence ABA levels by modulating endogenous zeatin riboside (ZR), IAA, ABA, and GA_3_ in floral organs, thereby affecting fruit set [99]. Brassinolide (BL) treatment enhances material conversion during seed development, increasing oil content—consistent with its known role in promoting oil synthesis in oilseed crops [100,101]. Li et al. (2023) reported that ABA and GA_3_ have antagonistic effects on squalene biosynthesis in *C. oleifera*: ABA (50 μM) upregulates *CoSQS* expression by 38%, while GA_3_ (50 μM) downregulates *CoSQS* expression by 25%. ABA treatment (100 μM) upregulated *CoHMGR_4* expression by 1.5-fold, whereas GA_3_ treatment (100 μM) downregulated it by 22% [102].

The AP2/ERF family mediates hormone-responsive terpenoid regulation. AP2/ERF genes improve target terpenoid yields in *Catharanthus roseus*, *Artemisia annua*, maize, and sweet orange [76,79,103,104,105]. LcERF19 correlates positively with *LcTPS42* expression and upregulates monoterpenoid-related genes [106]. Exogenous ethylene promotes endogenous ethylene biosynthesis during post-ripening in *Torreya grandis* and enhances squalene pathway gene expression [107]. Recent findings also indicate ethylene increases C18:2 and C18:3 levels in *C. oleifera* fruit [108]. 

### 4.3. Environmental Factors: External Regulatory Conditions

Light, temperature, and nutrient availability influence squalene biosynthesis by modulating plant metabolic activity. Terpenoid synthesis can be induced by herbivory, wounding, light, low temperatures, and other stresses [109]. Moderate light intensity (300–400 μmol·m^−2^·s^−1^) is most conducive to squalene accumulation in *C. oleifera* seedlings [8]. The *CoSQS* promoter harbors light-responsive elements, and different light wavelengths significantly affect squalene content in kernels [11]. HY5, a central light signaling integrator, transcriptionally regulates terpenoid biosynthesis across UV, red, and blue spectra [110,111]; in *Arabidopsis*, *AtHY5* negatively regulates squalene biosynthesis in a light-dependent manner [112]. PHY influences GA synthesis and ABA breakdown, impacting oil accumulation and seed maturation [113]. Light factors alone have been reported to increase *C. oleifera* seed oil content by 1% [114].

Zhang et al. (2024) found that blue light (450–490 nm) is the most effective wavelength for promoting squalene accumulation in *C. oleifera*, increasing squalene content by 48% compared to white light, which is related to the upregulation of *CoHY5* and *CoSQS* [115].

Drought stress significantly alters *C. oleifera* growth, enzyme secretion, stomatal morphology, and leaf osmotic regulators [116]. Expression levels of sterol biosynthesis genes—including *HMGS*, *HMGR*, *MK*, *DXS*, *IPPI*, *FPPS*, *SQS*, and *DWF1*—increase after drought treatment [59]. Light and temperature stresses may induce ABA and arginase expression, promoting upstream precursor synthesis to enhance oil, squalene, and sterol accumulation [117]. Additionally, *PvMYB1* activates *PvDGAT2* to promote TAG accumulation under heat stress in *Sacha inchi* [118].

Chen et al. (2023) found that moderate drought stress (soil water content of 50–60%) increases squalene content in *C. oleifera* seeds by 35%, mediated by the upregulation of *CoHMGR* and *CoSQS*. Moderate drought stress upregulated *CoDREB2A* expression by 2.1-fold, which further activated *CoDXS* and *CoDXR* expression [119].

## 5. Future Perspectives

Combining multi-omics technology, CRISPR/Cas9 genome editing, and synthetic biology strategies, subsequent research can further unravel the regulatory mechanisms underlying squalene biosynthesis and facilitate the breeding of high-squalene *C. oleifera* varieties. Concrete, feasible strategies are proposed as follows:

### 5.1. CRISPR/Cas9 Editing Targets

- Knock-out of negative regulators genes: The first exon of the repressor gene *CoWRKY72* can be edited via single gRNA. Previous gene-silencing results suggest that homozygous mutants may achieve a 40–60% rise in seed squalene content [63].
- Promoter modification: The endogenous *CoSQS* promoter with weak light and JA responsive elements can be substituted by artificially synthetized promoter containing multiple MBS and G-boxes motifs to sustain high-level gene expression. The CaMV 35S core promoter fused with four tandem MBS sequences derived from *CoFPS1* serves as an ideal candidate [59].
- Allele knock-in modification: Codon-optimized *CoSQS* can be integrated into the genome and driven by seed-specific promoter (e.g., *oleosin* promoter), so as to specifically elevate squalene accumulation in seeds.

### 5.2. Bottlenecks and Countermeasures of Genetic Transformation and Plant Regeneration

Agrobacterium-mediated transformation of *C. oleifera* faces prominent restrictions. The transformation efficiency of embryogenic callus is less than 5%, mainly restricted by low bacterial adhesion capacity and poor cell regeneration ability. In addition, the whole regeneration period lasts 8–12 months, which slows down experimental iteration. Moreover, genetic transformation is only applicable to limited cultivars, represented by Huashuo and Xianglin 1.

Corresponding solutions include introducing developmental regulator genes *WUS2* and *BBM* to improve regeneration efficiency, optimizing vacuum infiltration parameters and Silwet L-77 dosage, and constructing *C. oleifera*-specific Agrobacterium strains.

### 5.3. Synthetic Biology Approaches

- Co-express synergistic *CoMYB1*, *CoMYB44*, and *CoERF1* under one bidirectional promoter. Superposition effect is expected to boost squalene content by 2–3 times [59,60].
- Construct cytosolic bypass of MVA pathway by expressing truncated *HMGR* and fluorescent protein-tagged soluble SQS, enabling real-time dynamic monitoring of metabolic flux.

The above strategies can be implemented with existing experimental conditions, which greatly promotes the selective breeding of high-squalene *C. oleifera* cultivars. Improved varieties with superior oil quality and [59,60] medicinal value will accelerate the sustainable exploitation of this economically essential woody oil crop.

*Camellia oleifera* has a cultivation area exceeding 45 million mu nationwide. A 20–30% increase in squalene content could generate tens of billions of yuan in annual economic benefits for the tea oil industry, reduce dependence on squalene derived from sharks, and boost rural revitalization. This review lays a solid theoretical foundation for the genetic improvement of high-squalene *C. oleifera* germplasm.

## 6. Conclusions

This review systematically summarizes the research advances of squalene biosynthesis in *Camellia oleifera*. The MVA pathway acts as the predominant route for squalene production, whose biosynthesis is modulated by a sophisticated multi-layer regulatory network. This regulatory system covers multiple transcription factors, including WRKY, bHLH, and MYB families; endogenous phytohormones such as JA, ABA, and GA; and external environmental stimuli.

Four pivotal research findings are concluded in this work. First, *CoIDI* mediates material exchange and crosstalk between MVA and MEP pathways. Second, synergistic interactions among MYB transcription factors and antagonistic hormonal effects jointly govern metabolic flux. Third, post-translational modification exerts vital regulatory effects on *CoAACT* activity. Fourth, light and drought stresses impose pronounced impacts on squalene accumulation at the molecular level.

Current research still has notable limitations. Specifically, the spatiotemporal dynamics of transcription factor activity and metabolic flux during seed development remain poorly understood. In addition, epigenetic regulation of squalene synthetic genes has not been fully explored, and systematic metabolic flux analysis is still lacking.

## Figures and Tables

**Table 2:** Transcription factors involved in squalene biosynthesis in *Camellia oleifera* and their regulatory roles. All data are supported by direct experimental evidence from *C. oleifera*.

Transcription Factor	Target Genes	Regulated Pathway	Regulatory Effect	Experimental Evidence	Year
*CoWRKY15*	*CoSQS*	MVA (downstream)	Positive (1.6-fold squalene increase)	Y1H, OE in callus	2021, 2024 [11,62]
*CoWRKY72*	*CoSQS*	MVA (downstream)	Negative (silencing ↑ 45% squalene)	ChiP, silencing	2024 [63]
*CoMYC2*	*CoHMGR_4*	MVA (upstream)	Positive	Y1H, JA inducibility	2023, 2024 [64,65]
*CoMYB1*	*CoFPS1*, *CoHMGR_4*	MVA (middle/upstream)	Positive (1.3–1.8-fold FPP)	OE in seeds	2023 [59]
*CoMYB44*	*CoFPS1*, *CoIDI*	MVA (middle), crosstalk	Synergizes with *CoMYB1* (60% ↑ squalene)	Co-OE, Y2H	2025 [60]
*CoERF1*	*CoSQS*	MVA (downstream)	Synergizes with *CoMYB1* (2.3-fold ↑)	Y2H, Co-OE	2023 [59]
*CoDREB2A*	*CoDXS*, *CoDXR*	MEP (upstream)	Positive under drought (1.3-fold squalene)	OE in seedlings	2023 [58]

Note: ↑ represented that the expression was up-regulated.

## Data Availability

The original contributions presented in this study are included in the article. Further inquiries can be directed to the corresponding author.
